# Long-term recovery of canopy 3D structural diversity following wildfires in the world’s largest temperate woodland

**DOI:** 10.1098/rspb.2025.1095

**Published:** 2025-11-26

**Authors:** Beibei Zhang, Suzanne M. Prober, Alison O'Donnell, Carl R. Gosper, Fabian Fischer, Katherine Zdunic, Tommaso Jucker

**Affiliations:** ^1^ School of Biological Sciences, University of Bristol, Bristol BS8 1TQ, UK; ^2^ CSIRO Environment, Canberra ACT 2601, Australia; ^3^ CSIRO Environment, Waterford WA 6152, Australia; ^4^ Biodiversity and Conservation Science, Department of Biodiversity Conservation and Attractions, Kensington WA 6151, Australia

**Keywords:** canopy height, canopy gaps, disturbance, recovery, LiDAR, remote sensing, tree growth and mortality, wildfire

## Abstract

Wildfires play a major role in shaping the structure and dynamics of many woody ecosystems, with growing concerns that their frequency and intensity are increasing with climate change. However, we lack an understanding of how canopy structure recovers after wildfires, which limits our ability to forecast the long-term impacts of these disturbances on key ecosystem functions such as carbon storage and biodiversity. Using airborne laser scanning data acquired across a 450 year chronosequence of time since fire, we modelled the recovery trajectory of canopy 3D structural diversity across the largest temperate woodland on Earth in Western Australia. We found that canopy height, cover and heterogeneity recovered at varying rates and followed distinct trajectories. Canopies became taller, denser and more vertically homogeneous during the initial 100–150 years following fire. Subsequently, height growth plateaued while canopy cover continuously decreased for several centuries, leading to open and spatially heterogeneous structures in old-growth woodlands. The highly predictable nature of these structural recovery trajectories following wildfires allowed us to develop robust models for mapping stand age based on structural features. Our study paves the way for leveraging emerging remote sensing technologies to track ecosystem recovery from disturbance, thereby guiding management and restoration interventions at scale.

## 1. Introduction

Conserving, restoring and improving the management of woody ecosystems is widely recognized as an important nature-based solution for tackling the pressing climate and biodiversity crises we currently face [1–3]. Woody ecosystems are central to many of the United Nations Sustainable Development Goals as well as international initiatives such as the United Nations Decade for Ecosystem Restoration and the Bonn Challenge [4,5]. However, conservation and restoration efforts need to contend with the growing pressures on forests and woodlands from both human-driven and natural disturbances, such as droughts, storms, pest outbreaks and wildfires [1,6,7]—many of which are becoming increasingly frequent and severe due to climate change [8,9]. Understanding how key attributes of woody ecosystems recover from disturbance—such as their canopy structure, which directly constrains carbon storage and habitat suitability for biodiversity [10–14]—is critical for guiding restoration interventions and developing more realistic models of their dynamics [15,16]. Just as important, characterizing historic responses to disturbances is essential for establishing baselines to compare future recovery trajectories of woody ecosystems subjected to novel disturbance regimes [17–19].

However, tracking forest and woodland recovery from disturbance is inherently challenging for several reasons. One major issue is the spatial and temporal scales at which disturbance and recovery processes occur. Disturbances such as those caused by wildfires, storms, drought, pest and pathogen outbreaks can impact large areas and result in the death of entire tree cohorts [20–23]. These events are relatively infrequent, making them challenging to capture using permanent survey plots [24,25]. Additionally, characterizing subsequent recovery from these disturbances can be equally challenging, as these processes can take decades or even centuries to unfold [26]. In particular, growing evidence suggests that the recovery pathways forests and woodlands follow from disturbances are highly context dependent, varying enormously depending on factors such as climate, ecosystem type, species and their life-history traits, and the disturbance regime itself [27–30]. Taking wildfires as an example, in some ecosystems species have developed adaptations that enable them to either survive or quickly recover following fires (e.g. resprouting), while in others wildfires can completely reset the ecological stage [31,32]. In the latter cases, recovery depends on the presence of an adequate seedbank, seed dispersal, recruitment and the subsequent growth rates of regenerating trees, which will depend on the region’s climate and ecological processes such as competition and herbivory [33,34]. To complicate things further, various aspects of community structure and composition (e.g. aboveground biomass, community size structure, species diversity and traits) recover at different rates [28,35], making it difficult to build a comprehensive picture of the recovery trajectory of an ecosystem.

Remote sensing technologies offer an intuitive solution to several of the challenges of tracking ecosystem recovery from disturbance. Satellites have been used to capture the recovery trajectories of a wide range of ecosystems using both space-for-time approaches and time-series data [2,36,37]. For example, in open canopy ecosystems satellite data have revealed that vegetation greenness typically recovers to pre-fire levels within just a few years [38,39]. However, much of what can be observed from satellite spectral imagery (e.g. vegetation greenness and cover) only captures the short-term response of vegetation to fire, as these attributes are weak and indirect proxies of ecosystem properties related to vegetation structure and woody biomass [12]. In contrast, technologies like airborne laser scanning (ALS, or airborne LiDAR—light detection and ranging) generate highly detailed 3D models of entire vegetated landscapes which directly capture information relating to tree size structure, age and aboveground biomass [12,40,41]. These 3D models can also be used to infer attributes such as microclimate, canopy connectivity and deadwood abundance [42–44], all of which influence habitat suitability for many species [10,45]. Moreover, growing evidence suggests that the horizontal and vertical configuration of tree canopies, and their spatial organization across the landscape, shape their susceptibility to disturbance by wildfires, drought and wind [16,46,47]. By using ALS to map the long-term reorganization of canopy 3D structure following large-scale disturbances such as wildfires, we can therefore gain key insights into the long-term recovery potential of carbon stocks, biodiversity and overall ecosystem resilience [48,49].

**Figure 1.**
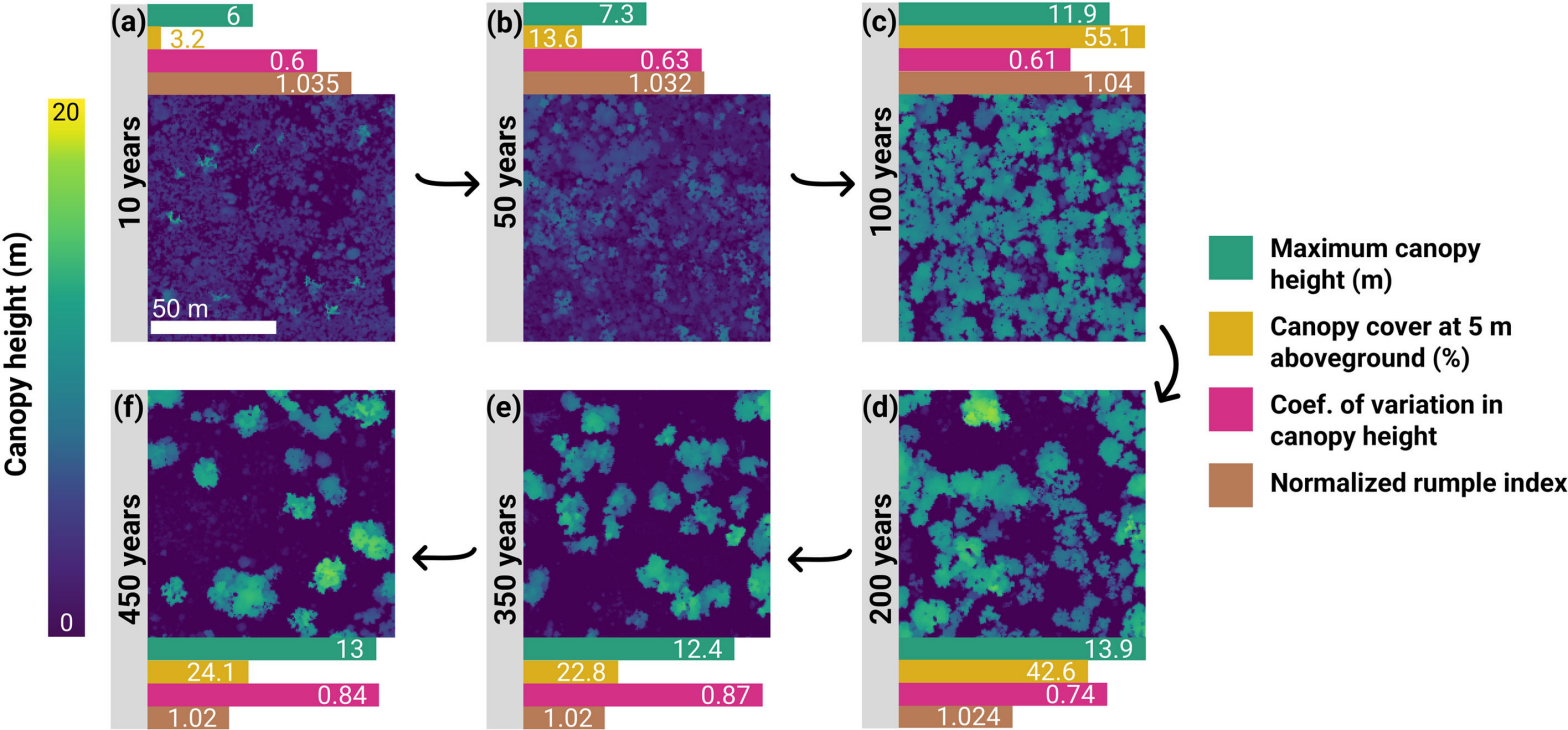
Illustration of how canopy 3D structure changes in the decades to centuries following stand-replacing fires in obligate-seeder eucalypt woodlands in the Great Western Woodlands. Each panel corresponds to a 100 × 100 m section of a canopy height model of plots that span a range of stand ages, from 10 to 450 years since the last stand-replacing fire. The horizontal bars show how each of the four canopy structural metrics explored in this study change across this chronosequence.

Here, we focus on Australia’s Great Western Woodlands (GWW) as a testbed for understanding how canopy 3D structure recovers over multiple centuries following wildfires. The GWW spans over 160 000 km^2^ and contains the largest extant temperate woodland ecosystem on Earth [40]. These woodlands are of unique conservation and cultural significance but face a growing threat from increased frequency and extent of wildfires [8,35,40,50,51]. They are dominated by obligate-seeder eucalypt species that are highly susceptible to wildfires, which are almost always stand-replacing. Following wildfires, field observations indicate that the woodlands undergo a developmental pathway that begins with dense, single-cohort stands that gradually thin out into open woodlands dominated by large, sparsely distributed trees [31,52]. To capture this recovery process, we acquired ALS data from 250 locations distributed across the GWW that span a 450 year chronosequence of time since the last stand replacing fire (figure 1). We used the ALS data to measure four complementary metrics that together describe the full spectrum of canopy 3D structure archetypes observed across the landscape [53]. We then used generalized additive models (GAMs) to describe the recovery pathways of each of these structural metrics and test how their trajectories vary in relation to rainfall. Finally, we used these models to test how accurately we could predict stand age based on canopy 3D structure. In doing so, we aimed to develop a tool for assessing broad spatial patterns of recovery across the region and guide management and restoration interventions in this biodiversity hotspot.

## 2. Methods

### (a) The Great Western Woodlands

Located in Australia’s southwest, the GWW are characterized by a semi-arid Mediterranean climate with an average annual rainfall of 200–400 mm and are distributed across an array of soil and bedrock formations [50]. Obligate-seeder woodlands are pervasive on valley floors and greenstone ranges, but absent from sandplains. They are dominated by a handful of eucalypt species that reach 15–25 m in height at maturity, the most common of which include *Eucalyptus salmonophloia*, *E. vittata*, *E. salubris* and *E. transcontinentalis* [54]. Wildfires are the dominant disturbance agent in the GWW and almost always result in complete stand-replacement [54], thereby driving woodland dynamics across the region.

This is because the obligate-seeder eucalypts that dominate the canopy are all highly sensitive to fires, lacking the lignotubers that allow other woody species to resprout following wildfires [54]. For example, low-intensity experimental surface fires have been shown to cause mortality in over 98% of *E. salubris* individuals [31], with even higher rates of mortality expected under wildfire conditions. Even in relatively more fire-tolerant species such as *E. salmonophloia*, only very few individuals survive low-intensity fires by resprouting from epicormic buds in the trunk [55].

Instead, obligate-seeder eucalypts rely on canopy-stored seed banks for regeneration following wildfires, with stand developmental dynamics spanning multiple centuries as trees are long-lived and slow-growing [31]. Post-fire successional dynamics are not associated with consistent shifts in the composition of canopy-forming tree species [31,56]. This is because even though individual trees can live for centuries [57] and produce seeds more or less continuously once they reach maturity [58], recruitment is limited by a lack of long-distance dispersal [56] and typically occurs only after disturbances like fire kill mature trees [55]. By contrast, understory shrubs and herbs with long-distance seed dispersal mechanisms tend to increase in diversity during succession [56], but contribute little to the 3D structure of the canopy. Collectively, these features make the GWW an ideal study system to characterize the long-term structural recovery trajectories of woodlands following stand-replacing disturbances using space-for-time approaches applied to chronosequence data.

### (b) Stand age chronosequence

To capture how canopy structure recovers following wildfires, we established a network of 250 field plots that span a broad chronosequence from 1 to around 450 years since the last stand-replacing fire (figure 1 and electronic supplementary material, figure S1 for plots distribution map). These plots were established between 2010 and 2021 and are distributed across the entire GWW region, covering a rainfall gradient from around 250 to 330 mm yr^-1^. In each plot, we used field data to quantify two attributes of woodland size structure that reflect stand development stage and aboveground biomass: basal area (m² ha⁻¹) and the density of living trees (trees ha⁻¹). For each plot, stand age was estimated using two approaches. For plots that burnt during the last 50 years, the year of the most recent stand-replacing fire was derived from the Department of Biodiversity, Conservation and Attractions fire history database (https://catalogue.data.wa.gov.au/dataset/dbca-fire-history), which includes digitized fire records obtained from a combination of aerial photos and Landsat imagery. This approach avoids overestimating the age of young stands in the unlikely event that one or more mature trees survive a fire. For plots with no history of fire in the past 50 years, stand age was estimated using a local tree size-age model developed using tree ring records [57].

### (c) Airborne laser scanning data acquisition and processing

We acquired ALS data across each of the 250 field plots between March and June of 2021 using a combination of both an airplane (226 plots) and an unoccupied aerial vehicle (UAV, 24 plots). The aircraft mission was led by the geospatial surveying company Aerometrex (https://aerometrex.com.au) using a Cessna 404 Titan mounted with a RIEGL VQ-780ii scanner. Flights were conducted at a height of 1100 m above ground level and a speed of 135 knots, resulting in point clouds with a mean pulse density of 22 pulses m^-2^ and a scan angle range of ± 33° (99% range). Point cloud data were georeferenced and combined by Aerometrex and provided in LAS format (i.e., a standard binary file format).

The UAV data were acquired using a DJI Matrice 600 Pro multi-rotor UAV mounted with a RIEGL miniVUX-1UAV scanner. The UAV was flown at 90 m above ground level at a speed of 7 m s^-1^. For each plot, scans were performed in two orthogonal directions with a 60 m distance between flightlines, resulting in point clouds with a mean pulse density of 50 pulses m^-2^ and a scan angle range of ± 50°. The trajectory of each flight was processed using POSPac UAV (Applanix) using RINEX data obtained from a locally operated Global Navigation Satellite System (GNSS) base station. Point clouds were then aligned and georeferenced using RiPROCESS software and exported in LAS format.

All subsequent data processing and analysis were conducted in R [59]. Specifically, we used a recently developed pipeline for generating canopy height models (CHMs) from ALS data that calls LAStools (https://lastools.github.io) through R [60]. This processing routine uses a locally adaptive spike-free algorithm to create 1 m resolution CHMs that are highly robust to differences in ALS pulse densities, scan angles, footprint sizes and laser power both among and within scans [60]. It was developed using data from Australia’s Terrestrial Ecosystem Research Network SuperSites (including the GWW) specifically for the purpose of allowing robust comparisons between CHMs generated from different sources of ALS data (e.g. UAV and airborne platforms) and has since been extensively tested on a global ALS database [61].

Prior to analysis, all CHMs were manually inspected and cropped to ensure they included no roads and only captured a single vegetation type. For each plot, the mean area (±1 s.d.) covered by the CHMs was 4.4 ± 2.0 ha (see electronic supplementary material, figure S2 for the size distribution of all plots). All CHMs used in this analysis are archived on the Global Canopy Atlas database [61].

### (d) Canopy structural metrics

We used the CHMs to characterize variation in woodland canopy structure across the 250 plots. Specifically, for each plot we used a combination of custom R functions [53] and the *terra* package [62] to calculate four structural metrics related to canopy height, openness and heterogeneity: (i) maximum canopy height (*H_max_*; 98% percentile of the CHM), (ii) canopy cover at 5 m aboveground (*Cover_5m_*; proportion of CHM pixels above 5 m, a threshold chosen to exclude standing dead wood after fire, although note that very similar results were obtained when using a 2 m height threshold; electronic supplementary material, figure S3), (iii) the coefficient of variation in canopy height (*H_cv_*; values bound between 0 and 1, with higher values indicating greater height heterogeneity; [63]), and (iv) a normalized rumple index (*Rumple_norm_*; a measure of rugosity defined as the ratio of canopy surface area to ground projected area divided by *H_max_*; [64]).

These four metrics were chosen as previous work has shown they capture independent axes of variation in canopy 3D structure in these ecosystems (electronic supplementary material, figure S4). Crucially, this work also showed that these four CHM-derived metrics can be calculated from different sources of ALS data (e.g. UAV and airborne platforms) without this affecting their values [53]. Consequently, our use of an ALS processing pipeline that generates robust CHMs and our careful choice of structural metrics allowed us to confidently combine ALS data acquired using different platforms in our study. Note that we did not include a measure of mean canopy height in our analysis, as this was tightly correlated with *Cover_5m_* (Pearson correlation coefficient, *r* = 0.92; electronic supplementary material, figure S3).

### (e) Modelling post-fire recovery trajectories of canopy structural metrics

We used GAMs to model the recovery trajectory of the four canopy structural metrics derived from ALS and the two ground-based metrics of stand structure (basal area and tree density) across the 450 year chronosequence of time since fire. GAMs were chosen as we expected canopy structural attributes to change nonlinearly during stand development, which GAMs capture using smoothing splines. In addition to stand age, models also incorporated the effects of plot size and mean annual rainfall on canopy structure. Rainfall is known to strongly influence rates of tree growth, mortality and regeneration [65,66], as well as tree allometry and height [47,67]. Rainfall estimates for each plot were obtained from the ERA5-Land dataset (https://www.ecmwf.int/en/era5-land), which are provided at 9 km^2^ resolution and cover the period between 1950 and 2021. Plot size was included in the models to account for the fact that three of the metrics (*H_max_*, *H_cv_* and *Rumple_norm_*) exhibited a degree of scale dependence, with their values being affected by the size of the plots (electronic supplementary material, figure S2).

GAMs for each of the six structural metrics were fitted using the *mgcv* R package with restricted maximum likelihood [68] and took the following form:


$$Y=f_{1}\left(Stand\;age\right)+f_{2}\left(Rainfall\right)+\;f_{3}\left(Plot\;size\right),$$

where *Y* is the response variable (basal area, tree density, *H_max_*, *H_cv_*, *Cover_5m_* or *Rumple_norm_*) and *f_1-3_* are the smooth functions of the three model predictors (stand age, rainfall and plot size). Tree density was log-transformed prior to model fitting to constrain its skewed distribution and improve the fit of the model. We used the adjusted *R*^2^ as a measure of explained variance and calculated annual rates of change for each response variable as the derivative of the response curve. Note that we chose not to include an interaction term between stand age and mean annual rainfall, as the two were not entirely independent (*r* = −0.57) and we lacked sufficient old-growth plots at the higher end of the rainfall gradient to test this interaction robustly (electronic supplementary material, figure S5).

### (f) Predicting stand age from airborne laser scanning-derived 3D structural attributes

To understand how well stand age can be predicted from canopy structural information derived from ALS data, we flipped the structure of the previous GAMs. Specifically, we built a series of increasingly complex GAMs where stand age was expressed as a function of either: (i) a single canopy structural metric (*H_max_*, *H_cv_*, *Cover_5m_* or *Rumple_norm_*), (ii) all possible two-way combinations of the four canopy structural metrics (six alternative GAMs) and (iii) all four structural metrics together. Rainfall and plot size were also included as predictors in all models to account for their influence on the relationship between canopy structure and age.

To robustly assess the predictive accuracy of these models, we split plots into training and validation datasets using a stratified random sampling approach that ensured the validation subset spanned the full range of stand ages. Specifically, the data were divided into 11 age classes of equal length (35 year intervals). Within each class, 90% of the data were randomly selected to fit the GAMs while the remaining 10% were reserved for model validation. This routine was repeated 500 times to generate robust estimates of model performance by comparing predicted and observed values in the validation subset on the basis of root mean square error (RMSE, in years) and the proportion of explained variance (*R*^2^). RMSE and *R*^2^ values were then compared across models to identify those with the best predictive power. Models were assessed for spatial autocorrelation using semivariograms, which revealed no evidence of spatial patterning in the residuals.

## 3. Results

### (a) Woodland canopy structure changes dramatically during stand development

**Figure 2.**
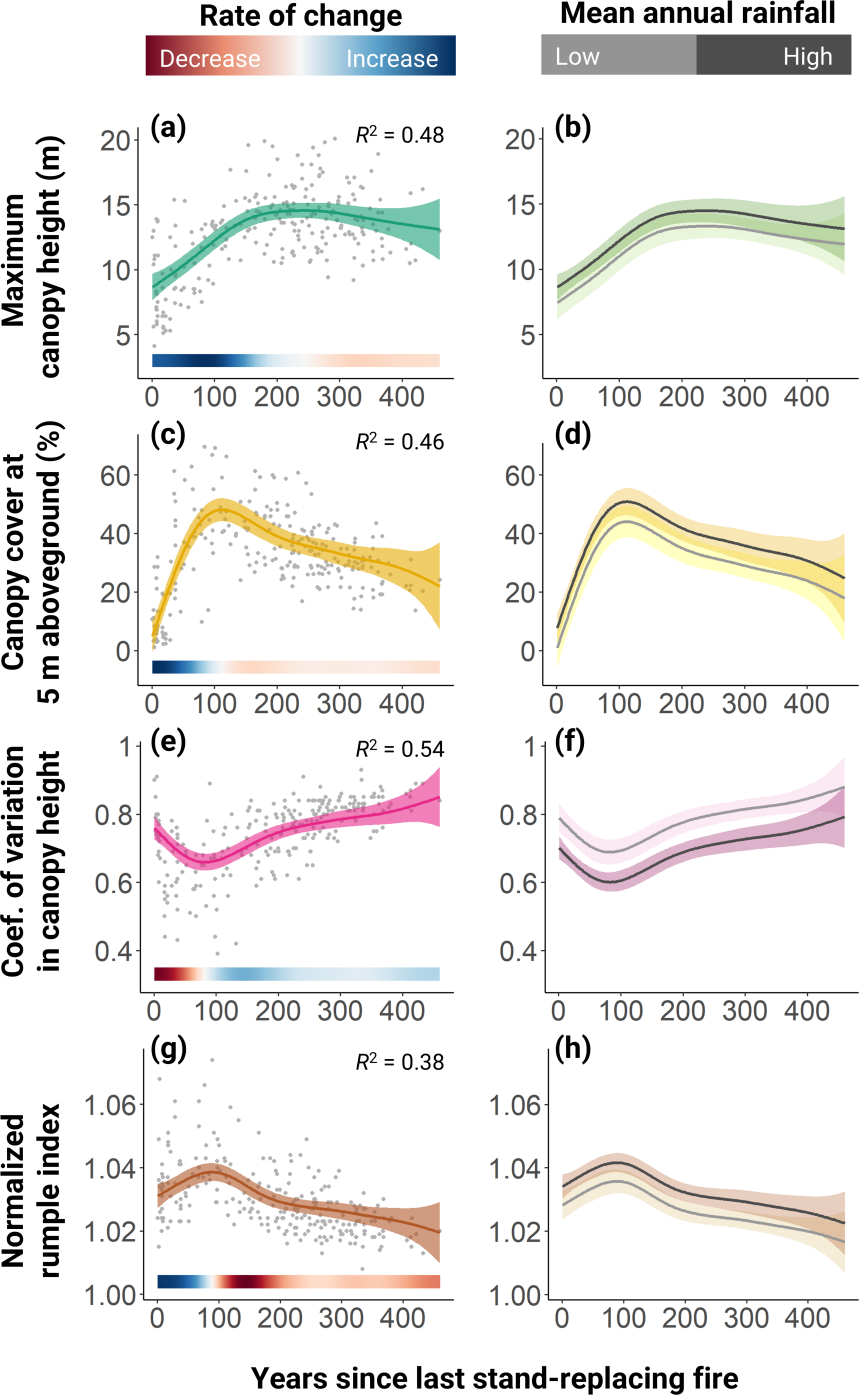
Recovery trajectories of the four canopy structural metrics derived from ALS data following stand-replacing fires. Panels on the left-hand side show the distribution of the data across the 250 field plots (grey points) with the fit of a GAM superimposed (shaded region corresponds to 95% CIs). The bars along the bottom of the panels indicate the annual rate of change of each structural metric calculated as the derivative of the fitted GAM. The *R*^2^ of each model is reported in the top right corner (electronic supplementary material, table S1). The *R*^2^ values also account for the influence of rainfall on each structural metric, the effects of which are displayed in the panels on the right-hand side. Specifically, dark grey lines correspond to predictions for woodlands at the 95^th^ percentile of the rainfall distribution (302 mm yr^-1^), while light grey lines show those for woodlands at the 5^th^ percentile of the rainfall gradient (260 mm yr^-1^).

Woodland structure varied dramatically across the 250 field plots (figure 1), with different facets of canopy structure following distinct nonlinear recovery trajectories following stand-replacing fires (figure 2). Overall, GAMs explained 31% and 87% of the variation in basal area and tree density among plots, respectively, and 38%–54% of the variation in the four ALS-derived canopy structural metrics (electronic supplementary material, table S1). Across all structural metrics, stand age emerged as the primary driver of variability among plots (figure 2 and electronic supplementary material, table S1). Despite having distinct recovery trajectories, each metric exhibited the fastest rate of change during the early to mid-stages of stand development (initial 75–150 years post-fire; figure 2 and electronic supplementary material, figure S6).

Tree density declined continuously during stand development, from over 4000 trees ha^-1^ in newly regenerating stands to around 20 trees ha^-1^ in old-growth plots that had not burned for more than 400 years (electronic supplementary material, figure S6b). By contrast, stand basal area increased progressively during the first 150–170 years after fire, reaching a peak of around 15 m^2^ ha^-1^ at a rate of 1.1 m^2^ ha^-1^ decade^-1^. After this point, basal area began declining gradually until it reached around 10 m^2^ ha^-1^ in stands older than 400 years (electronic supplementary material, figure S6a).

These changes in tree size structure were mirrored by the ones in canopy 3D structure. In particular, *H_max_* increased steadily for around 150 years post-fire at a rate of 0.9 m decade^-1^, reaching a peak of around 15 m on average before plateauing in older stands (figure 2a). *Cover_5m_* initially followed a similar trajectory, peaking at 48% on average at around 120 years post-fire, but then decreased substantially after that at a rate of around 0.8% decade^-1^ until it reached around 20% on average in very old stands (figure 2c). *Rumple_norm_* exhibited a similar trend to canopy cover, peaking soon after stand-replacing fires and then declining more or less continuously during stand development in a similar way to tree density (figure 2g). *H_cv_* instead exhibited the opposite pattern, starting high in recently burned stands before decreasing to a low point at around 75 years post-fire, only to then progressively increase again across the remainder of the chronosequence (figure 2e).

### (b) Rainfall influences the recovery trajectory of woodlands after fire

While stand age was the predominant driver of variation in woodland structure (electronic supplementary material, table S1), all four ALS-derived metrics of canopy 3D structure also varied significantly across the rainfall gradient captured by our plot network (figure 2). In particular, both canopy height and cover were higher at the wetter end of the rainfall gradient, with peak values of *H_max_* being around 1.2 m taller (figure 2b) and *Cover_5m_* around 7% higher (figure 2d). Similarly, *Rumple_norm_* was greater in areas with higher mean annual rainfall (figure 2h). By contrast, *H_cv_* was around 10% lower in plots at the wetter end of the spectrum (figure 2f).

### (c) Woodland age estimation from airborne laser scanning-derived structural metrics

**Figure 3.**
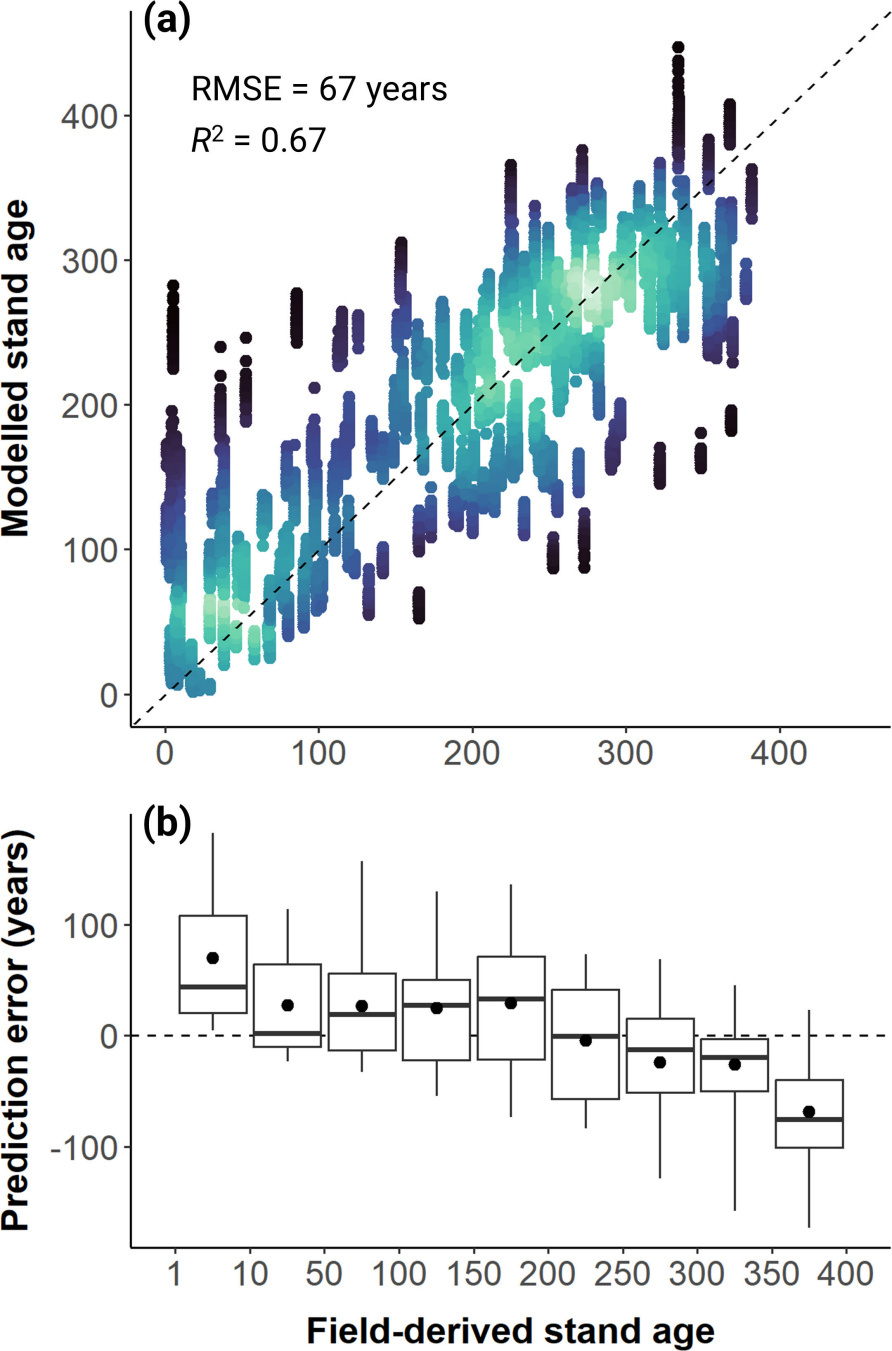
Predictive accuracy of the best-fitting generalized additive model (GAM) used to estimate stand age based on the four ALS-derived metrics of canopy structure (see electronic supplementary material, table S2 for details on the model structure and comparison to other GAMs). The scatterplot (a) compares field-derived and modelled estimates of stand age, with the dashed line corresponding to a 1:1 relationship and the colour gradient reflecting the density of overlapping points, from low (black) to high (light green). Boxplots (b) show how prediction errors (defined as predicted—observed stand age) vary among different woodland age classes.

GAMs, fit using different combinations of the four canopy structural metrics, were able to provide relatively accurate predictions of stand age (electronic supplementary material, table S2 and figure 3). *H_max_* emerged as the single best predictor of stand age (*R^2^* = 0.62 and RMSE = 71 years). However, we found that a GAM fitted with all four structural metrics together generally provided a better fit to the data compared with any single or two-way combination of structural metrics (*R^2^* = 0.67 and RMSE = 67 years; figure 3a), even though in some cases gains in predictive power were marginal (electronic supplementary material, table S2). Moreover, even for the best performing model we did find that stand age estimates for both recently burned and very old woodlands were systematically biased (figure 3b). Specifically, very young woodlands were sometimes mistaken for very old ones based on their 3D canopy structure. Similarly, for mature stands over 350 years old, the model underestimated stand age by an average of 68 years.

## 4. Discussion

Our study provides the first comprehensive assessment of how canopy structural diversity recovers following wildfires in the largest temperate woodland ecosystem on Earth [36,39,69,70]. We show that different axes of canopy 3D structure related to height, openness and spatial patterning exhibit characteristic recovery patterns that evolve over multiple centuries after stand-replacing fires. Our work provides key insights into the resilience of this globally important biodiversity hotspot to shifting disturbance regimes and future climate change, with direct implications for guiding restoration efforts, biodiversity conservation, wildfire management and safeguarding carbon stored across the region [40,52,71]. More broadly, by integrating chronosequence data with high-resolution ALS surveys, our study provides a general framework for tracking post-disturbance dynamics at broad spatial scales and a wide range of ecosystems [2,72]. In doing so, it improves our ability to predict how forest canopies respond to disturbance and provides a benchmark to assess their future recovery [73].

### (a) The long arc of structural recovery following wildfires

Canopy structural attributes directly related to tree height, density, size distribution and spatial organization all displayed clear and distinctive multi-century recovery pathways following wildfires in the GWW (figures 1 and 2). Broadly speaking, we identified two distinct phases in these stand development trajectories. Post-fire recruitment initially led to the formation of increasingly dense, vertically homogenous stands that grew progressively in stature until reaching a plateau at around 150 years of age. After this, we observed a clear inflection point in the system. Canopy cover peaked at around 120 years after the fire and then began steadily decreasing for over three centuries. This gradual opening of the canopy led to increased vertical and horizontal heterogeneity in canopy structure, as captured by an increase in *H_cv_*. It was also reflected in a continual decline in *Rumple_norm_* in stands older than around 75 years. As a measure of canopy surface area scaled by *H_max_*, *Rumple_norm_* essentially captures the number of peaks in the CHM and is therefore closely tied to the density of large canopy trees in this system. Taken together, these trends illustrate how in these semi-arid woodlands it takes at least 300 years, if not more, for canopy structural diversity to fully recover after wildfires and reach what can be considered an old-growth state.

Reconstructing changes in canopy structure over time provides several important insights into the demographic processes that shape the developmental pathways of these woodlands [74]. Of particular interest is the very marked trend of woodland canopy thinning that unfolds as stands mature. This pattern suggests that despite increasingly large gaps created by the mortality of mature trees in stands older than 100–200 years [41], only a limited amount of understory regeneration occurs in this system [31,75]. As seedlings in these canopy openings would not be light limited, a lack of understory reinitiation could be linked to nutrient depletion, as nitrogen, phosphorus and other micronutrients essential for plant growth can become locked up in woody vegetation over time and require fire to be released back to soil [76,77]. Competition for water with mature trees that have much more extensive root networks could also limit seedling establishment in gaps [78].

Capturing the timing and rate of canopy thinning is also crucial to understand the future risk of wildfire in the GWW. The more open and sparse canopies of old-growth woodlands are known to act as a natural barrier to fires, dampening their spread by limiting the amount and spatial continuity of ground fuel [75,79]. But as wildfires in the region become increasingly frequent and severe [40,80], there is a real risk that fire return intervals will simply become too short to allow woodlands to recover to this more open structural state, locking them into a fire trap [81]. This would have major implications both for the ability of these ecosystems to sequester and store carbon—which is substantial despite the arid climate [41,52]—and provide habitat for the unique biodiversity they support [13,51].

### (b) Water availability constrains woodland 3D structure across the Great Western Woodlands

In addition to fire, water availability is also an important driver of vegetation 3D structure in the GWW (figure 2), mirroring well-known regional and global shifts in forest cover and tree height along rainfall gradients [74,82–84]. The GWW are an ecosystem moulded by their semi-arid climate, which limits maximum tree height to under 25 m and leads to open canopy woodlands dominated by very slow-growing trees that take several centuries to reach maturity [31,40]. Our results show that even modest differences in mean annual rainfall (<50 mm yr^-1^) across the region leave a clear fingerprint on forest structure, with canopies becoming shorter, more open and spatially heterogeneous as the climate becomes increasingly arid.

This is concerning in the context of climate change, as southwestern Australia is one of the fastest-drying regions of the planet [85]. Winter rainfall has declined by around 20% since the 1970s, with aridity increasing noticeably since the early 2000s [86]. This suggests that as the climate continues to warm, the GWW will become shorter, sparser, store less carbon aboveground and potentially even transition to a new state characterized by much lower tree cover. Additionally, climate change may also pose an indirect threat to these woodlands by facilitating the spread of perennial invasive grasses such as *Cenchrus ciliaris* (buffel grass). While buffel grass is currently rare in the GWW, having only been recorded at a handful of roadside locations [87], the region is expected to become more climatically suitable for its spread [50,88]. Unlike annual grasses such as *Pentameris airoides*, which contribute little to fuel accumulation, buffel grass can substantially increase fuel loads and fire frequency [89], potentially further disrupting canopy structural recovery in the future.

While the broad distribution of our plot network allowed us to quantify the general influence of rainfall on canopy structure across the GWW region, we were not able to test if the shape of the post-fire recovery trajectory is modified by water availability. For example, in drier areas we might expect lower rates of seedling establishment and growth and higher adult tree mortality [90], slowing down initial post-fire recovery [91,92] and speeding up canopy thinning later on. The issue we faced is that in our dataset, woodland age and rainfall are not independent of each other, making it challenging to statistically test how they interact [93]. Specifically, woodland age and rainfall were negatively correlated and all sites estimated to be older than 300 years were in areas with less than 300 mm yr^-1^ of rainfall (electronic supplementary material, figure S5). This is partly a limitation of our plot network, which was primarily designed to capture a gradient in stand age as opposed to rainfall. But it likely also reflects the fact that in the GWW fire frequency generally increases with rainfall because of changes in canopy structure related to water availability. As our data show, higher rainfall generally promotes the development of denser canopies and understories, which in turn enhances vertical and horizonal fuel continuity (e.g. ladder fuels) and therefore increases the risk of fire spread [35,79]. By contrast, the sparser and more open canopies of dry woodlands act as a natural fire barrier, increasing the likelihood that woodlands persist to very old ages in these drier climates [40].

### (c) Large-area woodland age mapping for targeted conservation, restoration and wildfire management

The highly predictable nature of the structural recovery trajectories explored in our analysis allowed us to build models that produce reliable estimates of stand age based on canopy 3D structure (electronic supplementary material, table S2 and figure 3). These models have the potential to play a big role in guiding targeted wildfire management strategies at local and regional scales, as they can be used to generate high-resolution (4 ha) maps of stand age from ALS data. Continuous stand age maps would also help prioritize on-ground woodland conservation and restoration interventions that aim to enhance landscape resilience to changing wildfire regimes and avoid fire traps. In this regard, our work goes well beyond previous efforts to map woodland age using remote sensing, which had been limited to coarse-resolution (1 km^2^) products that simply distinguished between recently burned, intermediate aged and mature woodlands older than 120 years [40].

Age estimates derived from our models are not perfect (figure 3b). Systematic underestimation of time since fire in the older age classes is likely caused by dilution bias arising from uncertainty in the estimates of stand age. A more concerning issue is the tendency of the models to sometimes confuse very young stands with very old ones. A likely explanation is that recently burned woodlands actually share some of the same structural features as old-growth ones. Not only do they have low vegetation cover, but because many trees die standing after fire and take years or even decades to fall over and decompose [94], recently burned stands have high structural heterogeneity. This is captured very clearly by the *H_cv_* metric (figure 2e), which is initially high in recently burned stands and then progressively decreases over the first 50–75 years of stand development as snags slowly fall down and the regenerating tree cohort grows in stature.

Looking ahead, there are several ways in which estimates of stand age could be refined. One option would be to use the ALS data to develop targeted metrics that better distinguish between old and recently burned woodlands, such as ones that capture the crown size of mature individual trees [41]. A distinctive feature of old-growth stands is the presence of sparsely distributed trees with large crowns that exceed 20 m in width, whereas snags in recently burned plots have little or no visible crowns (figure 1). Alternatively, existing wildfire maps generated from Landsat imagery that extend back to the early 1970s could be used to cross-reference stand age estimates and correct those predicted to be old-growth in areas known to have burned in the past 50 years. More ambitiously, woodland age maps generated from ALS could be combined with Landsat or Sentinel archives to train machine learning algorithms that estimate stand age from spectral satellite data. This would not only enable high-resolution mapping of present-day woodland age across the entire GWW but would also allow historical reconstructions of woodland age prior to recent wildfires [13]. This would provide key spatial information on fire return intervals, which is essential to forecasting how the unique ecosystems of the GWW will respond to ongoing changes in fire regimes.

### (d) Towards a global picture of forest structural recovery from disturbance

Our study provides a blueprint for future efforts to integrate field and remote sensing data to track how the 3D structure of forests, woodlands and savannas recovers in the aftermath of disturbance [95]. Recent years have seen a rapid rise in the use of ALS technologies to map ecosystems in 3D with increasing precision and detail [14,44]. What was once a specialized and expensive technology is now increasingly available to a wide range of researchers, with UAV laser scanning in particular growing rapidly in use [53]. Moreover, there have been several recent efforts to aggregate existing ALS datasets into harmonized databases, including the newly launched Global Canopy Atlas initiative which brings together ALS data acquired across thousands of forest landscapes all over the world [61]. In addition to ALS, today researchers also have access to huge collections of laser scanning data acquired from space, in particular those generated by the Global Ecosystem Dynamics Investigation (GEDI) mission [40,96]. In parallel, there have also been big advancements in using satellite archives to map annual forest disturbances at regional and global scales going back as far as the 1980s [97,98]. These maps not only provide information on when and where a given disturbance event occurred, but increasingly also record how severe it was and what caused it [97–99]. Combining these two complimentary data streams using chronosequence approaches like the one we developed here would substantially advance our empirical understanding of how and why recovery from disturbance varies across different woody ecosystems [2,96]. Moreover, these data also provide a unique opportunity for longitudinal studies that actively track dynamics over time [1,41,96,100], as opposed to relying on space-for-time substitutions (and all the assumptions these make [101]) to infer recovery trajectories. Beyond the numerous policy, management, conservation and restoration applications of this research, this work would also provide invaluable empirical data to train and validate new generations of dynamic global vegetation models (DGVMs) and individual-based forest simulators that better capture the structure and dynamics of Earth’s woody ecosystems [95,102,103].

## Data Availability

All ALS-derived CHMs used in this analysis are archived on the Global Canopy Atlas database [61]. R code to replicate the calculation of the structural metrics described in this paper was developed as part of a previous publication and is publicly available on Zenodo: [104]. Plot-level structural metrics and fire chronosequence data, as well as R code for replicating the results and figures of this study are publicly available on Zenodo: [105]. Mean annual rainfall data were obtained from the ERA5-land dataset produced by the European Centre for Medium-Range Weather Forecasts (https://www.ecmwf.int/en/era5-land). Supplementary material is available online [106].
